# Capicua suppresses colorectal cancer progression via repression of ETV4 expression

**DOI:** 10.1186/s12935-020-1111-8

**Published:** 2020-02-05

**Authors:** Jeon-Soo Lee, Eunjeong Kim, Jongeun Lee, Donghyo Kim, Hyeongjoo Kim, Chang-Jin Kim, Sanguk Kim, Dongjun Jeong, Yoontae Lee

**Affiliations:** 1 Department of Life Sciences, Pohang University of Science and Technology, Pohang, Gyeongbuk Republic of Korea; 2 Division of Integrative Bioscience and Biotechnology, Pohang University of Science and Technology, Pohang, Gyeongbuk Republic of Korea; 3 Soonchunhyang Medical Science Research Institute, Soonchunhyang University, Cheonan, Chungnam Republic of Korea; 4 Department of Pathology, College of Medicine, Soonchunhyang University, Room 601, 31 Soonchunhyang 6gil, Dongnam-gu, Cheonan, Chungnam 31151 Republic of Korea; 5 POSTECH Biotech Center, Room 388, 77 Cheongam-Ro, Nam-Gu, Pohang, Gyeongbuk 37673 Republic of Korea

**Keywords:** Colorectal cancer, Capicua (CIC), Polyoma enhancer activator 3 (PEA3), ETS translocation variant 4 (ETV4)

## Abstract

**Background:**

Although major driver gene mutations have been identified, the complex molecular heterogeneity of colorectal cancer (CRC) remains unclear. Capicua (CIC) functions as a tumor suppressor in various types of cancers; however, its role in CRC progression has not been examined.

**Methods:**

Databases for gene expression profile in CRC patient samples were used to evaluate the association of the levels of *CIC* and *Polyoma enhancer activator 3* (*PEA3*) group genes (*ETS translocation variant 1* (*ETV1*), *ETV4*, and *ETV5*), the best-characterized CIC targets in terms of CIC functions, with clinicopathological features of CRC. CIC and ETV4 protein levels were also examined in CRC patient tissue samples. Gain- and loss-of function experiments in cell lines and mouse xenograft models were performed to investigate regulatory functions of CIC and ETV4 in CRC cell growth and invasion. qRT-PCR and western blot analyses were performed to verify the CIC regulation of ETV4 expression in CRC cells. Rescue experiments were conducted using siRNA against *ETV4* and CIC-deficient CRC cell lines.

**Results:**

CIC expression was decreased in the tissue samples of CRC patients. Cell invasion, migration, and proliferation were enhanced in CIC-deficient CRC cells and suppressed in CIC-overexpressing cells. Among *PEA3* group genes, *ETV4* levels were most dramatically upregulated and inversely correlated with the CIC levels in CRC patient samples. Furthermore, derepression of *ETV4* was more prominent in CIC-deficient CRC cells, when compared with that observed for *ETV1* and *ETV5*. The enhanced cell proliferative and invasive capabilities in CIC-deficient CRC cells were completely recovered by knockdown of *ETV4*.

**Conclusion:**

Collectively, the CIC-ETV4 axis is not only a key module that controls CRC progression but also a novel therapeutic and/or diagnostic target for CRC.

## Background

Of the 36 various types of cancers diagnosed world-wide, colorectal cancer (CRC) ranks third among the most frequently occurring cancers and second in terms of cancer mortalities [1]. Common genetic alterations responsible for the development and progression of CRC include inactivation of the tumor suppressors *Adenomatosis polyposis coli* (*APC*) (~ 70%) and *TP53* (~ 60%) and mutational activation of *KRAS* (~ 40%) [2–7]. For the treatment of CRC, targeted therapy drugs such as bevacizumab and cetuximab, which are inhibitors of angiogenesis and the epidermal growth factor receptor (EGFR) pathway, respectively, have been actively developed [8]. However, these inhibitors cannot be used for the effective treatment of all CRC patients. Therefore, additional therapeutic strategies for the treatment of CRC must be developed.

Capicua (CIC) is a transcriptional repressor containing a high mobility group (HMG) box domain and a C-terminal motif that are evolutionarily conserved from *Caenorhabditis elegans* to humans [9–14]. Through the HMG box and C-terminal domains, CIC recognizes specific octameric DNA sequences (5′-T(G/C)AATG(A/G)(A/G)-3′) to regulate the expression of its target genes [12, 15, 16]. There are two main isoforms of CIC, the short (CIC-S) and long (CIC-L) form, which are distinguished by their amino-terminal regions [17, 18]. It is known that CIC is regulated by extracellular signal–regulated kinase (ERK), which is a downstream kinase of the RAS/RAF/MEK signaling cascade. Activation of the MAPK pathway (RAS/RAF/MEK/ERK) results in phosphorylation of CIC, and this ultimately leads to degradation or cytoplasmic localization of CIC [19–21]. CIC controls several essential processes including cell proliferation and tissue patterning in *Drosophila* [13, 22, 23]. In mammals, CIC is required for lung alveolarization, liver homeostasis, brain development and function, and immune cell homeostasis [24–28].

Accumulating evidence indicates that CIC functions as a tumor suppressor in various types of cancers. Previous studies have identified numerous *CIC* mutations in patients suffering from various types of cancers, including soft tissue, brain, lung, gastric, prostate, and breast cancers [9, 29–32]. Additionally, chromosomal translocations that generate the CIC-DUX4 chimeric form have been identified in Ewing-like sarcomas [9, 33–35]. Either mutations in or loss of CIC can promote cancer progression via upregulating the expression of *PEA3* group genes (*ETV1/ER81*, *ETV4/PEA3*, and *ETV5/ERM*), the best characterized and reliable CIC target genes [9, 32, 36, 37]. The PEA3 group factors are known as an oncogenic transcription factor, because the overexpression of these transcription factors promotes cancer cell proliferation and metastasis via activating the transcription of a subset of genes related to control of cell division and migration, such as matrix metalloprotease (MMP), vascular endothelial growth factor (VEGF), and telomerase reverse transcriptase (TERT) [38]. Several *CIC* mutations were found in the CRC patient samples (6 out of 74 samples) [39], and it is therefore conceivable that CIC may also be involved in the regulation of CRC progression. Regardless, the exact role of CIC in the suppression of CRC progression and the CIC target genes involved in this process remain to be investigated.

In this study, we examined the association of CIC and PEA3 group transcription factors with CRC clinicopathology by conducting analyses of the TCGA dataset and tissue samples derived from CRC patients. We also investigated the molecular basis underlying CIC-mediated regulation of CRC progression through the use of CRC cell lines and mouse xenograft models. Our study identifies the CIC-ETV4 axis as a key molecular module that controls CRC progression.

## Materials and methods

### Cell culture

HCT116 (ATCC_CCL-247™) and HT29 (ATCC_HTB-38™) colorectal cancer cells were cultured in DMEM (Welgene, Gyeongsan, Republic of Korea) containing 10% FBS (Welgene, Gyeongsan, Republic of Korea) and 1% penicillin/streptomycin (Gibco, MA, USA). Cells were incubated at 37 °C in a 5% CO_2_ incubator.

### Mice

Male BALB/C nude mice (5-week-old) were purchased from OrientBio (Seongnam, Republic of Korea) and were subjected to acclimatization for 1 week. They were then used for the in vivo tumor formation assay. Mice were fed standard rodent chow and water ad libitum and maintained in a specific pathogen-free animal facility under standard 12 h light/12 h dark cycle. All experimental procedures of animal studies followed the guidelines and regulations approved by the POSTECH Institutional Animal Care and Use Committee (IACUC).

### Human tissue samples

Human tissue samples were obtained from Soonchunhyang University Hospital (Cheonan, Republic of Korea). The colon tissue samples from 13 patients with CRC were used in this study. Informed consent was obtained from all patients. All procedures were approved by the Soonchunhyang University Hospital Institutional Review Board (SCHCA 2018-07-061-003).

### Generation of viruses and stable cell lines

*ETV4* shRNA and *CIC* sgRNA cassettes were cloned into MSCV-LTRmiR30-PIG (LMP) and lentiCRISPR v2 plasmids, respectively, according to the manufacturer’s manuals. HCT116 and HT29 CRC cells were infected with viral supernatants in the presence of polybrene (Sigma-Aldrich, MO, USA). After 24–48 h, the cells were selected using 2 µg/ml of puromycin (Gibco, MA, USA) for 48 h. For overexpression of CIC-S and ETV4, the cloned pHAGE-FLAG-CIC-S, pHAGE-ETV4, and pHAGE control plasmids were used. The lentivirus production process was described previously [36]. Viral supernatants were collected at 48 h post-transfection and were used to infect the HCT116 or HT29 cells for 3 sequential days. The cells were used for further biochemical assays as specified in each experiment.

### siRNA transfection

*ETV4* siRNA (siETV4) was purchased from Bioneer (Daejun, Republic of Korea). The sequences are as follows: siETV4 sense; 5′- GAGGAAUUCAGCUCAGCUUdTdT -3′, and antisense; 5′- AAGCUGAGCUGAAUUCCUCdTdT -3′. One day prior to transfection, 1 × 10^5^ cells were plated in 60 mm plates. After 24 h, the cells were transfected with 120 pmol of siRNA duplexes using Dharmafect 1, according to the manufacturer’s instructions. After 72 h, the cells were used for further biochemical assays as specified in each experiment.

### qRT-PCR

Total RNA was extracted using RiboEX (GeneAll, Seoul, Republic of Korea). cDNA was synthesized using a GoScript™ Reverse Transcript kit (Promega, WI, USA), according to the manufacturer’s instructions. SYBR Green PCR Mixture (Toyobo, NY, USA) was used for qRT-PCR analysis. Expression data were acquired using a StepOnePlus™ Real-Time PCR System (Applied Biosystems, CA, USA). Expression levels of each target were calculated using the 2^−ΔΔCt^ method and were presented as relative mRNA expression. The sequences of primers used for qRT-PCR were previously described [37].

### Cell lysis and immunoblotting

Cells were harvested and lysed in RIPA buffer (50 mM Tris (pH 7.4), 150 mM NaCl, 0.5% sodium deoxycholate, 0.1% SDS, and 1% Triton X-100) containing complete protease inhibitor cocktail tablets (Roche, Basel, Switzerland) by sonication. The lysates of CRC patients’ tissue samples were also prepared by sonication in RIPA buffer. The concentration of cell proteins was determined via the BCA assay. Western blot analysis was performed as described previously [25]. Generation of rabbit polyclonal anti-CIC antibody was previously described [25]. Anti-ETV4 (10684-1-AP) antibody was purchased from Proteintech (IL, USA). Anti-β-ACTIN (sc-47778) antibody was purchased from Santa-Cruz Biotechnology (TX, USA). HRP-conjugated secondary antibody was purchased from Pierce Thermo Scientific (MA, USA). The western blot images were obtained using ImageQuant LAS 500 (GE Healthcare Life Science, PA, USA).

### Cell growth assay

Stably infected cells (7 × 10^3^ cells) were seeded into each well of 24-well plates. The cells were trypsinized and stained with Trypan Blue (Sigma-Aldrich, MO, USA). The number of viable cells was counted using a hemocytometer every day for 4 days. For cell growth assays of siRNA-treated *CIC* knockout HCT116 or HT29 cells, 7 × 10^3^ cells were seeded into 24-well plates 1 day before transfection, and then siRNAs were transfected using Dharmafect 1 (Dharmacon, CO, USA) and set as day “0”. The cells were trypsinized and stained with Trypan Blue. The number of viable cells was counted using a hemocytometer every day for 4 days.

### In vitro migration and invasion assay

A 24-well trans-well plate (8-µm pore size, SPL, Pocheon, Republic of Korea) was used to measure the migratory and invasive abilities of each cell line. For trans-well migration assays, 5 × 10^4^ cells were plated in the top chamber lined with a non-coated membrane. The inserts were cultured in a well of 10% FBS containing media and were incubated for 6 h. They were then removed, washed with PBS, stained with formalin/0.1% crystal violet solution, and analyzed under a ZEISS Axioplan2 microscope. Multiple 5–10 images per insert were acquired, and the average counts were calculated. For invasion assays, chamber inserts were coated with 16 µl/ml of Matrigel (BD Biosciences, MA, USA) with DMEM/F12 media (Gibco, MA, USA) and dried overnight under sterile conditions. Next, 1 × 10^5^ cells were plated in the top chamber. The inserts were cultured in a well of 10% FBS-containing media and incubated for 48 h. The same staining method used in the migration assay was applied.

### In vivo tumor growth assay

For xenograft tumor growth assays, control and CIC KO cells (5 × 10^6^ cells) were subcutaneously injected into the posterior flank of 6-week-old male BALB/C nude mice. Seven days after inoculation, the tumor size was measured every week for 12–13 weeks. The tumor volume was calculated as 1/2 × (largest diameter) × (smallest diameter)^2^.

### Tissue microarray and immunohistochemistry

The colorectal cancer tissue microarray (CO2085b) was purchased from Biomax (MD, USA). Formalin-fixed paraffin-embedded specimens were deparaffinized and stained with rabbit polyclonal anti-ETV4 antibody (1:500 dilution). Each sample stained with anti-ETV4 antibody was scored as negative (−), weak (+), or strong (++) according to the staining intensity. These scores were determined independently by two pathologists in a blinded manner. Tissue samples from 9 CRC patients were provided by Soonchumhyang University Hospital (Cheonan, Republic of Korea). Formalin-fixed paraffin-embedded specimens were deparaffinized, and the antigens were retrieved via a citrate-buffered (pH 6.0) solution method. After blocking endogenous peroxidase activity, immunohistochemistry of CIC and ETV4 was performed using a VECTASTAIN Elite ABC HRP Kit (Vector Labs, CA, USA) according to the manufacturer’s instruction. Specimens were stained with home-made rabbit polyclonal anti-CIC antibody (1:500 dilution) [25] or anti-ETV4 antibody (1:500 dilution). The color reaction was performed using a DAB kit (Vector Labs, CA, USA). Then, the sections were counterstained with Mayer’s hematoxylin, dehydrated, and mounted. Images were acquired under an OLYMPUS BX41 microscope and analyzed by SPOT Basic image capture software.

### TCGA database analysis

Gene expression data from colorectal cancer and normal cells (mRNA, normalized RNAseq FPKM-UQ, July 2014) were retrieved from the TCGA database (provisional) using cBioPortal for cancer genomics during the diagnosed period from 1998 to 2013. Gene expression data were available for 453 CRC patients. Expression levels were log2 transformed. Clinical data including the tumor stage were downloaded from the TCGA portal in July 2014. Tumor stages were defined using the latest version of the American Joint Committee on cancer code at the time of diagnosis. Major tumor stages (I, II, III, or IV) were investigated for differences in gene expression. Expression levels of *CIC* and *PEA3* group genes after normalization were compared among the tumor stages. *P* values were calculated using a Mann–Whitney U test comparing the expression values in the patient samples at each tumor stages. The detailed clinical and pathological characteristics of CRC patients in TCGA database are listed in Additional file 1: Table S2.

### Statistical analysis

For statistical analysis, all experiments were performed more than thrice independently. Data are presented as mean ± standard error. The quantitative data were compared between groups using the Student’s *t* test (two-tailed, two-sample unequal variance). A value of *P* < 0.05 was considered to be statistically significant.

## Results

### Association of CIC with CRC

**Fig. 1:**
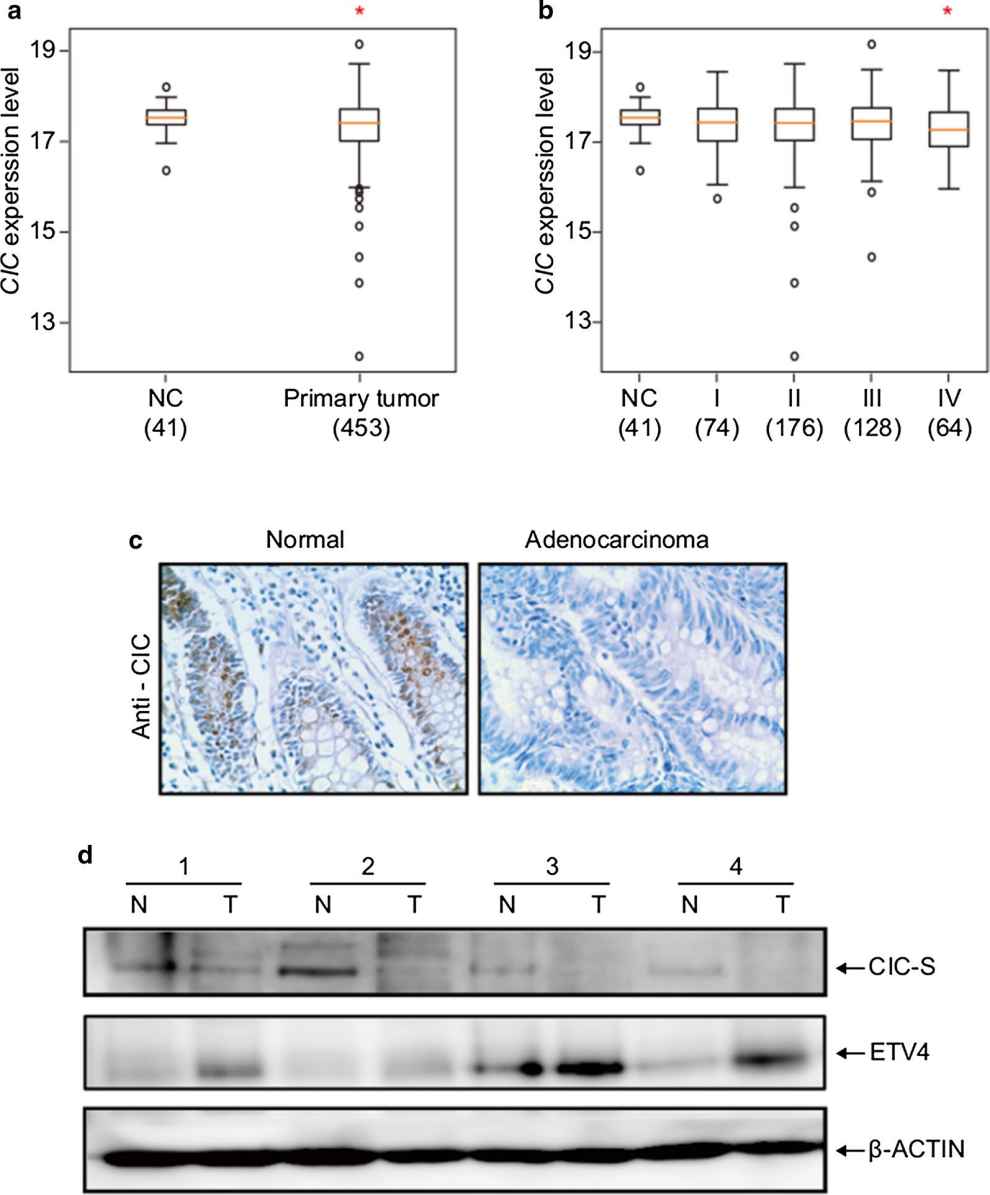
Loss of CIC in colorectal cancer. **a** Analysis of the TCGA dataset for *CIC* mRNA levels in normal colon (NC) and primary colorectal tumor samples. The numbers in parentheses indicate the number of subjects in each group. *P < 0.05. **b** Analysis of the TCGA dataset for *CIC* mRNA levels in normal colon (NC) and CRC samples of four different clinicopathological stages (I, II, III, and IV). The numbers in parentheses indicate the number of subjects in each group. *P < 0.05. **c** Analysis of CIC protein levels in normal colon and CRC tissues by immunohistochemistry. **d** Western blot analysis for CIC and ETV4 levels in normal colon (N) and CRC (T) samples from the same patient with CRC. Samples from four CRC patients were subjected to this experiment

To gain insight into the association between CIC and CRC, we searched for *CIC* mutations and expression changes in the CRC patient samples using public cancer databases. Several mutations in the *CIC* gene have been identified from CRC patient samples according to the information from the cBioPortal database [40] (Additional file 2: Fig. S1a). Moreover, CRC ranked fourth among the 27 different types of cancers in terms of the number of *CIC* mutations identified in each tumor type (Additional file 1: Table S1). Analyses of the datasets for CRC patients from The Cancer Genome Atlas (TCGA) and Catalogue of Somatic Mutations in Cancer (COSMIC) databases revealed that *CIC* levels were slightly decreased in the colorectal tumor samples compared with the normal tissues (Fig. 1a and b, Additional file 1: Table S2, and Additional file 2: Fig. S1b). Considering that CIC expression is robustly regulated by the RAS/MAPK signaling pathway at posttranslational levels [12, 32, 37], we examined the CIC protein levels in CRC tissue samples. Notably, the CIC expression was dramatically decreased in the CRC regions compared with that in the normal tissue area (Fig. 1c, Additional file 1: Table S3, and Additional file 3: Fig. S2). This result was confirmed by western blot analysis (Fig. 1d and Additional file 1: Table S4). Collectively, these data suggest that loss of CIC function is potentially associated with progression of CRC.

### CIC suppresses CRC cell growth, invasion, and migration

**Fig. 2:**
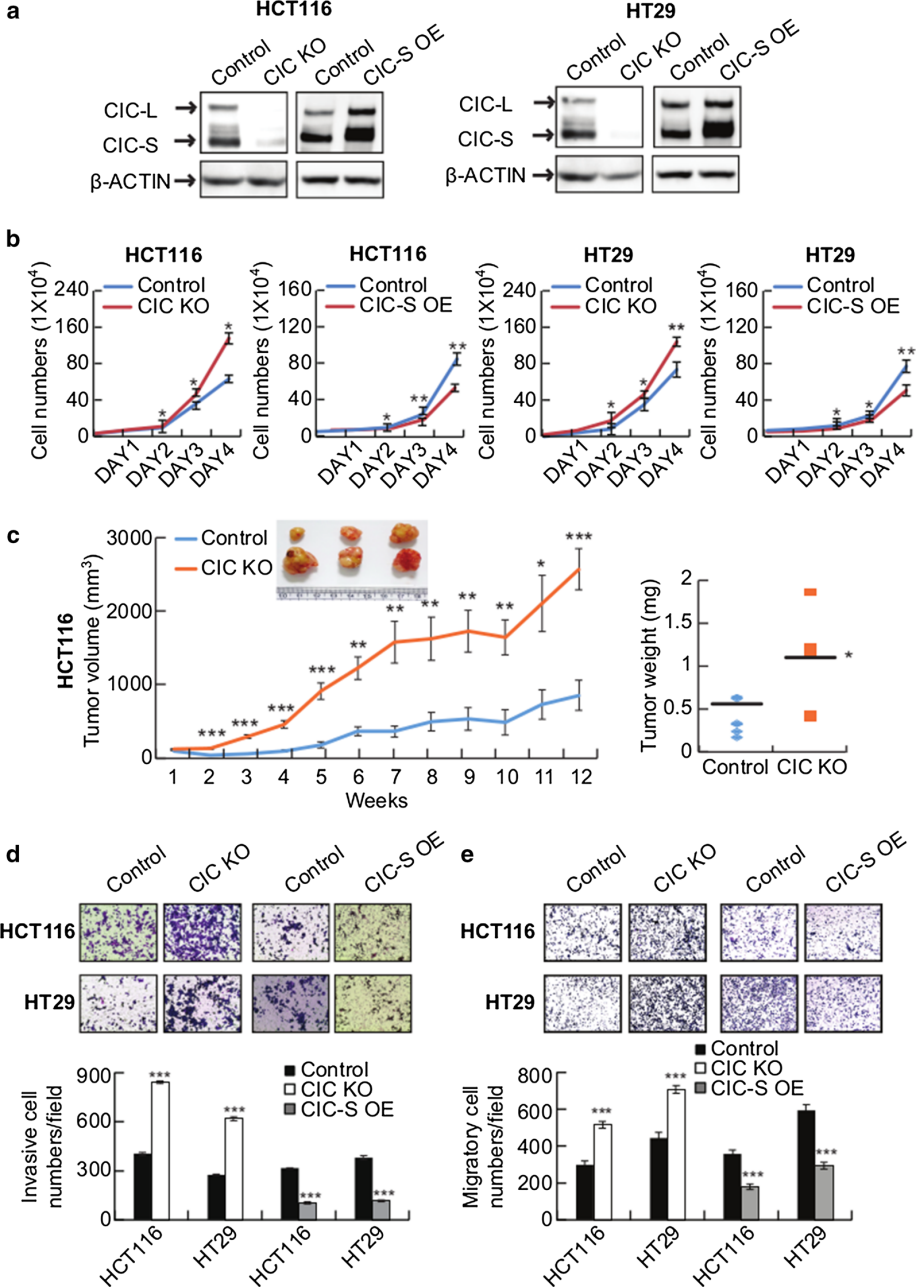
CIC suppresses CRC progression. **a** Western blot analysis presenting the overexpression and knockout of CIC in HCT116 and HT29 CRC cells. **b** Cell growth assay of the CIC-S-overexpressing and CIC-deficient (CIC KO) CRC cells. **c** In vivo subcutaneous tumor growth curves of control and CIC KO HCT116 cells. *n* = 7 per each group. The inset image is a representative image of xenograft tumors dissected from the mice after the last measurement of tumor size. The right panel is a graph for average weights of the dissected tumors. **d**, **e** Matrigel invasion **(d)** and trans-well migration **(e)** assay of control, CIC KO, and CIC-overexpressing CRC cells. The bottom panels are bar graphs for quantification of cell invasiveness **(d)** and cell migration **(e)**, respectively. Three independent experiments were performed. All error bars indicate s.e.m. *P < 0.05, **P < 0.01, and ***P < 0.001

To determine that CIC regulates CRC progression, we examined the cell proliferative, invasive, and migratory capabilities in CRC cells by varying the expression of CIC. We prepared CIC-deficient (CIC KO) cells in two different CRC cell lines (HCT116 and HT29) using *CIC*-targeted (exon4 of *CIC* gene) CRISPR-Cas9 system. Moreover, we generated CRC cells that overexpress CIC-S by a serial infection of lentivirus expressing CIC-S. Loss or overexpression of CIC expression was confirmed via immunoblotting (Fig. 2a). CIC deficiency promoted cell proliferation in both CRC cell lines, whereas the forced expression of CIC-S suppressed it (Fig. 2b). We confirmed these results in vivo using xenograft mouse models. We subcutaneously transplanted the control and CIC KO HCT116 cells into left and right posterior flank of the same BALB/c athymic mice, respectively, and measured the tumor volumes every week. The CIC KO cells grew more rapidly and formed larger tumor mass than the control cells (Fig. 2c). Furthermore, we tested the invasive and migratory capacities of CIC-deficient or CIC-overexpressing CRC cells. The invasive and migratory capabilities were negatively regulated by CIC in the CRC cells: there were more invaded and migrated cells in CIC KO CRC cells, whereas less in CIC-overexpressing cells (Figs. 2d, e). Collectively, these findings indicate that CIC suppresses CRC progression.

### ETV4 is most relevant to CRC progression among PEA3 group transcription factors

**Fig. 3:**
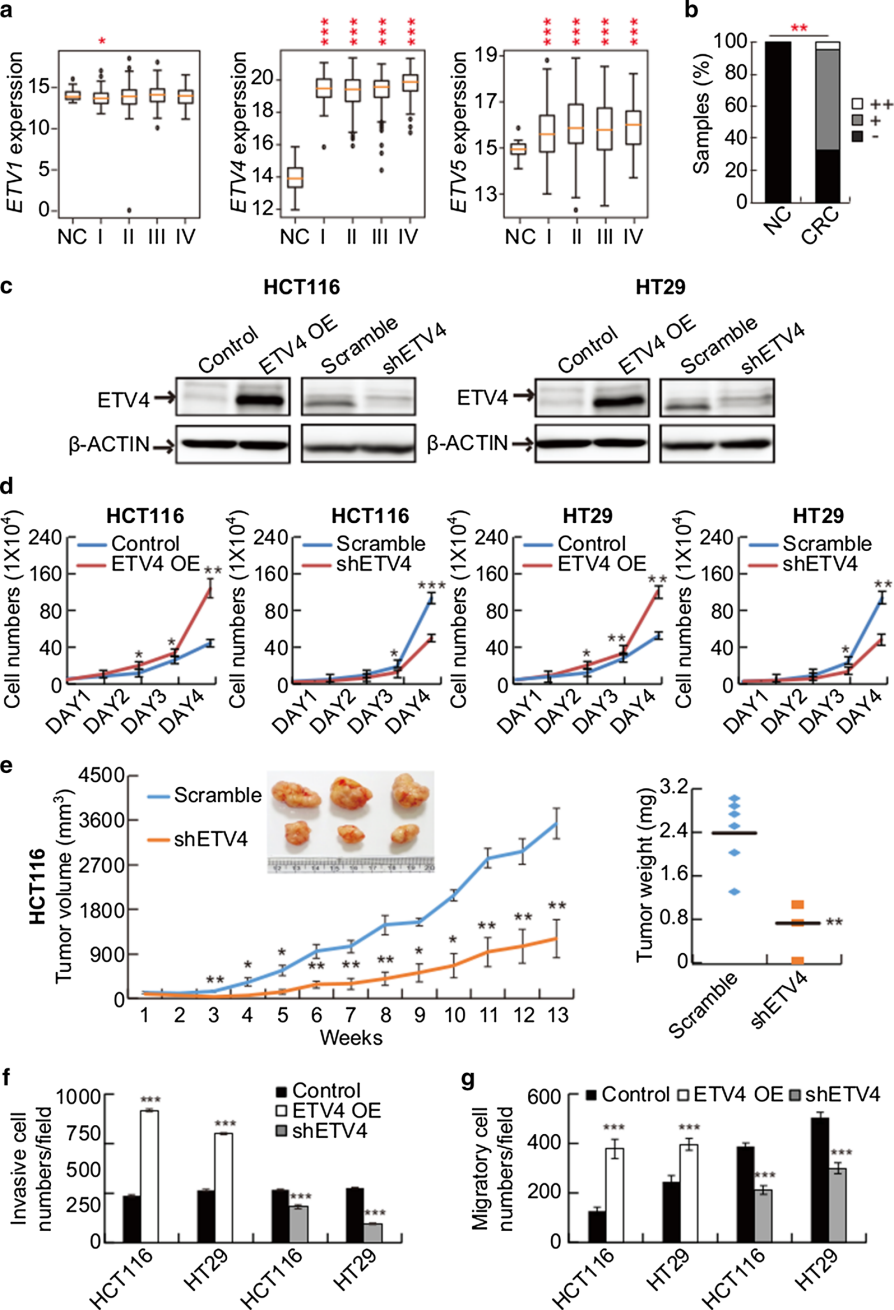
ETV4 promotes CRC progression. **a** Analysis of the TCGA dataset for levels of *ETV1, ETV4*, and *ETV5* in normal colon (NC) and CRC samples of four different clinicopathological stages (I, II, III, and IV). **b** The graph for the proportion of normal colon (*n* = 20) and CRC (*n* = 188) tissue samples with different ETV4 expression scores (−, +, ++). The CRC tissue microarray was subjected to immunohistochemical analysis of ETV4 protein levels. **c** Western blot analyses of ETV4 levels in ETV4-overexpressing (ETV4 OE) and ETV4-knockdown (shETV4) CRC cells (HCT116 and HT29). **d** Cell proliferation assay of ETV4-overexpressing (ETV4 OE) and ETV4-knockdown (shETV4) CRC cells. **e** In vivo subcutaneous tumor growth curves of control (scramble) and ETV4-knockdown (shETV4) HCT116 cells. *n* = 6 per each group. The inset image is a representative image of xenograft tumors dissected from the mice after the last measurement of tumor size. The right panel represents a graph for average weights of the dissected tumors. **f**, **g** Matrigel invasion (**f**) and trans-well migration (**g**) assay of control, ETV4-overexpressing, and ETV4-knockdown CRC cells. The bottom panels represent the bar graph for quantification of cell invasiveness **(f)** and cell migration **(g)**, respectively. Three independent experiments were performed. All error bars indicate s.e.m. *P < 0.05, **P < 0.01, and ***P < 0.001

Considering that *PEA3* group genes are known as oncogenic transcription factors [38] as well as direct target genes of CIC [9, 24, 31, 41–43], we examined the relevant factors for CRC progression among these three genes. The TCGA dataset analysis revealed that *ETV4* and *ETV5* were significantly upregulated across all stages of CRC (Fig. 3a), and that *ETV4* expression was notably increased in the CRC samples compared with that in the normal colon tissues among the *PEA3* group genes (Fig. 3a), consistent with previous findings that overexpression of ETV4 was frequently observed in samples from patients with CRC [39, 44, 45]. We confirmed the overexpression of ETV4 proteins in CRC tissue samples via immunohistochemistry (CRC tissue microarray and 10 CRC patients’ samples; Fig. 3b, Additional file 1: Table S3, and Additional file 4: Fig. S3) as well as western blot analysis (4 CRC patients’ samples; Fig. 1d, Additional file 1: Table S4).

To verify that ETV4 has a tumor promoting activity in the CRC cells, we generated ETV4-overexpressing and ETV4-knockdown CRC cell lines (Fig. 3c). Overexpression of ETV4 promoted proliferation of CRC cells, whereas knockdown of ETV4 suppressed it (Fig. 3d). This result was also confirmed in xenograft mouse models (Fig. 3e). Moreover, increased and decreased invasive and migratory capabilities were observed in ETV4-overexpressing and ETV4-knockdown CRC cell lines, respectively (Fig. 3f, g). Collectively, these results suggest that ETV4 might be most critically associated with CRC progression among the PEA3 group transcription factors.

### CIC deficiency promotes CRC progression via *ETV4* derepression

**Fig. 4:**
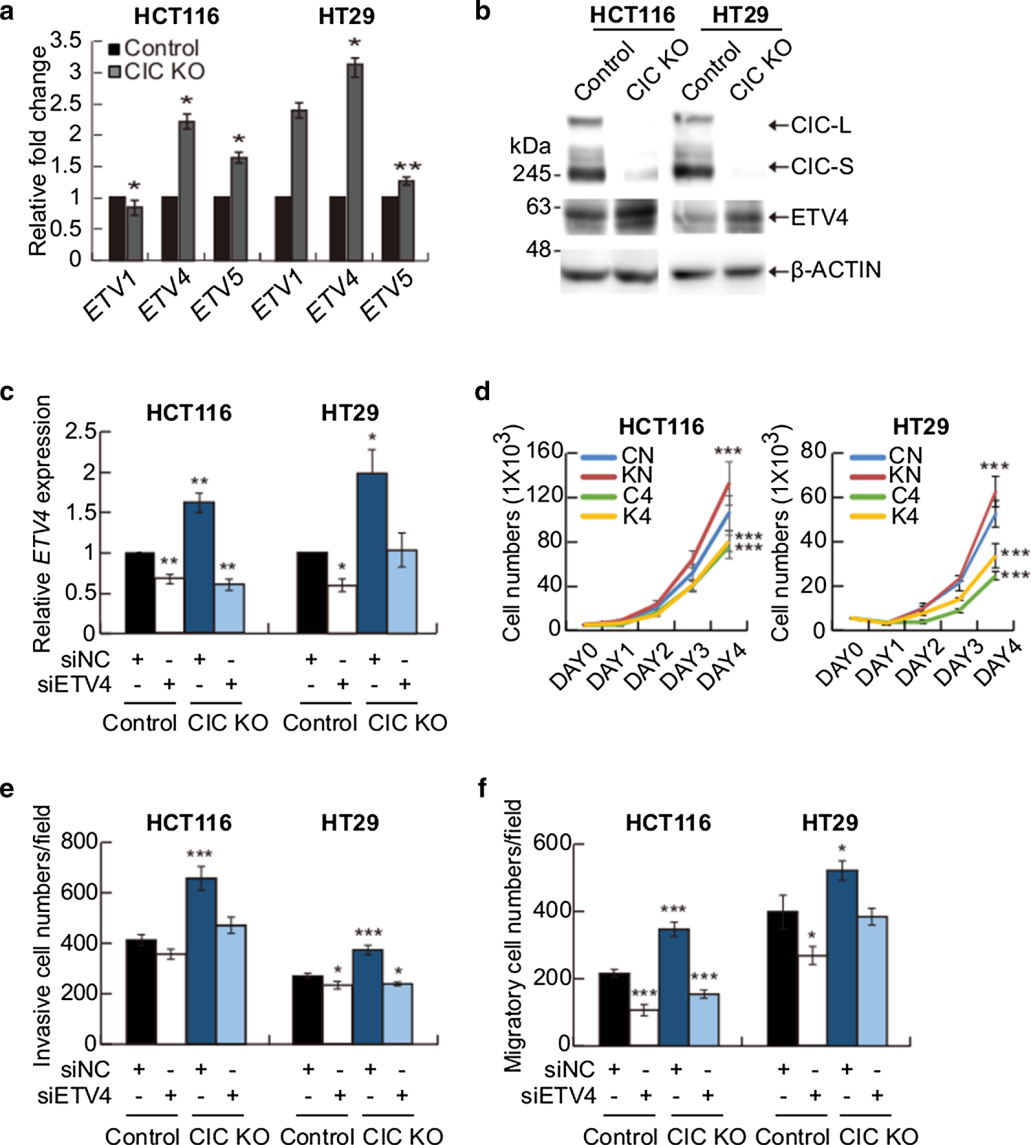
CIC deficiency-mediated promotion of CRC progression is due to derepression of *ETV4*. **a** qRT-PCR analysis of *PEA3* group gene expression levels in control and CIC-deficient (CIC KO) CRC cells (HCT116 and HT29). **b** Western blot analysis presenting upregulation of ETV4 expression in CIC KO CRC cells. **c** qRT-PCR analysis for *ETV4* mRNA levels in control and CIC KO CRC cells transfected with either control (siNC) or *ETV4* siRNA (siETV4). **d–f** Cell growth assay (**d**), Matrigel invasion assay (**e**), and trans-well migration assay (**f**) of control and *CIC* knockout CRC cells treated with either siNC or siETV4. Three independent experiments were performed. All error bars indicate s.e.m. *P < 0.05, **P < 0.01, and ***P < 0.001

To determine the transcription factor-target gene relationship between CIC and *PEA3* group genes in CRC cells, we investigated alterations in expression of *PEA3* group genes by loss of CIC in CRC cells. Among the three genes, *ETV4* and *ETV5* were derepressed, and *ETV4* had the highest fold increase in both CIC KO HCT116 and HT29 cells (Fig. 4a), suggesting that the regulation of *ETV4* expression might be most considerably dependent on CIC in CRC cells compared with those of *ETV1* and *ETV5*. The derepression of ETV4 was also confirmed at protein levels in CIC KO CRC cells (Fig. 4b). Consistent with these results, we have also observed that there was an inverse correlation between CIC and ETV4 expression in normal colon (N) and CRC (T) samples from the same patient with CRC (Fig. 1d).

We finally determined whether the increased cell proliferation, invasion, and migration in the CIC-deficient CRC cells were due to the derepression of *ETV4*. To this end, we transfected the control and CIC KO CRC cells with either control siRNA or siRNA against *ETV4* (siETV4). Treatment with siETV4 relieved the upregulation of *ETV4* levels in CIC KO CRC cells (Fig. 4c). We then examined cell proliferation, invasion, and migration in this set of CRC cells. Knockdown of *ETV4* substantially blocked the CIC deficiency-mediated promotion of cell proliferation (Fig. 4d), invasion (Fig. 4e), and migration (Fig. 4f), demonstrating that the CIC-ETV4 axis regulates CRC progression.

## Discussion

Previous studies have shown that CIC functions as a tumor suppressor in various types of cancers, such as brain, lung, gastric, prostate, and liver cancers [30–32, 36, 37]. In most cases, CIC deficiency promotes cancer progression via derepression of *PEA3* group genes, and the degree of derepression of each member of the *PEA3* group genes is variable among cancer types: *ETV5* is the most significantly and dramatically upregulated in CIC-deficient prostate cancer cells [36], while *ETV4* is upregulated in liver cancer cells [37]. Our findings demonstrate that CIC functions as a tumor suppressor in CRC cells and highlight ETV4, among the PEA3 group transcription factors, as a strong promoter of cancer progression and as a critical target of CIC in the context of CRC.

Analyses of the TCGA dataset and tissue samples from CRC patients revealed that CIC expression was more prominently reduced in CRC patients at the protein level than it was at the mRNA level. Somatic mutations of *KRAS* occur in over 40% of sporadic CRC, and abnormal activation of mutated KRAS affects the activation of the downstream molecules [46, 47]. As activation of the RAS/MAPK signaling pathway suppresses CIC activity via degradation or cytoplasmic retention of CIC in *Drosophila melanogaster* and mammals [20, 32, 48], the decreased expression of CIC in samples obtained from CRC patients may result from enhanced activity of MAPKs. Reduced expression of CIC proteins was also observed in tissue samples from patients with other types of cancers, such as prostate and liver cancers [36, 37]. Therefore, a decrease in CIC levels may be one of the key features occurring in the process of cancer progression in various types of cancers that exhibit hyperactivation of RAS/MAPK signaling.

Alterations in several essential developmental signaling pathways including WNT, NOTCH, and Sonic hedgehog (SHH) are known to be associated with CRC progression [49] Among these, oncogenic mutations in the WNT pathway genes are prevalent in CRC. Mutations inactivating *APC* are found in 70–80% of CRCs, and these are believed to initiate malignant transformation of the colorectal epithelial cells [7, 49]. However, the majority of APC mutant colon epithelial tumors are benign and never progress to CRC, suggesting that other genetic alterations are required for the development of WNT signaling-mutant colon epithelia into CRC. Given this, it is conceivable that *CIC* might be a gene whose loss or inactivating mutations drive CRC development and progression via collaboration with the WNT pathway. Consistent with this, ETV4 stabilizes β-catenin, a key transcription factor mediating WNT signaling, to promote tumor aggressiveness in gastrointestinal stromal tumors [50]. In future studies, it will be interesting to explore if and how the CIC-ETV4 axis cross-talks with the major signaling pathways such as WNT signaling that are altered in CRC cells.

## Conclusions

This is the first study to demonstrate that CIC functions as a tumor suppressor in CRC cells. Our findings also highlight ETV4 as a strong promoter of cancer progression as well as a critical target of CIC in the context of CRC. In conclusion, the CIC-ETV4 axis is a key molecular module that controls CRC progression.

## Supplementary information

**Additional file 1: Table S1.** List of *CIC* mutations in various types of cancers from the International Cancer Genome Consortium (ICGC) database. **Table S2.** Clinical and pathological characteristics of the COAD patients in TCGA. **Table S3.** List of 9 CRC patient samples (for Fig. 1c, Additional file 3: Fig. S2 and Additional file 4: Fig. S3). CRC patient samples were provided by Soonchunhyang University Hospital (South Korea). **Table S4.** List of 4 CRC patients (for Fig. 1d). CRC patient samples were provided by Soonchunhyang University Hospital (South Korea).

**Additional file 2: Figure S1.** Mutations and expression levels of *CIC* in CRC patient samples. a Schematic illustration of the location of point mutations in *CIC* identified from patients with colorectal cancer. The image was captured from the cBioPortal database. b Analyses of two different datasets (GSE5206 and GSE20916) from COSMIC database for *CIC* mRNA levels in normal and colorectal tumor tissues. The numbers in parentheses indicate the number of subjects in each group.

**Additional file 3: Figure S2.** Reduced expression of CIC in colorectal tumors. Immunohistochemical staining of tissue samples from patients with CRC using anti-CIC antibody. CIC expression is substantially decreased in the tumor areas compared with that in the normal colon areas.

**Additional file 4: Figure S3.** Increased expression ETV4 in colorectal tumors. Immunohistochemical staining of tissue samples from patients with CRC using anti-ETV4 antibody. ETV4 expression is dramatically increased in the tumor areas compared with that in the normal colon areas.

## Data Availability

All data generated or analyzed during this study are available from the corresponding author on reasonable request.

## Abbreviations

CRC: colorectal cancer
CIC: Capicua
PEA3: polyoma enhancer activator 3
ETV4: ETS translocation variant 4
EGFR: epidermal growth factor receptor
HMG: high mobility group
TCGA: The Cancer Genome Atlas
COSMIC: Catalogue of Somatic Mutations in Cancer
CIC KO: CIC-deficient
MAPK: mitogen activated protein kinase
