# Supplementary material for: Capicua suppresses colorectal cancer progression via repression of ETV4 expression
Source: Cancer Cell Int. 2020 Feb 5;20:42. doi: 10.1186/s12935-020-1111-8 (PMC7003492; doi:10.1186/s12935-020-1111-8)
Supplement: Supplementary file 1 — Additional file 1: Table S1. List of CIC mutations in various types of cancers from the International Cancer Genome Consortium (ICGC) database. Table S2. Clinical and pathological characteristics of the COAD patients in TCGA. Table S3. List of 9 CRC patient samples (for Fig. 1c, Additional file 3: Fig. S2 and Additional file 4: Fig. S3). CRC patient samples were provided by Soonchunhyang University Hospital (South Korea). Table S4. List of 4 CRC patients (for Fig. 1d). CRC patient samples were provided by Soonchunhyang University Hospital (South Korea). [file 12935_2020_1111_MOESM1_ESM.docx]

**Additional file 1: Table S1. List of *CIC* mutations in various types of cancers from the International Cancer Genome Consortium (ICGC) database.**

| **Site** | **Tumor Type** | **# Donors affected** | **# Mutations** |
| --- | --- | --- | --- |
| Liver | Liver cancer | 83/1495 (5.55%) | 152 |
| Skin | Skin cancer | 83/518 (16%) | 138 |
| Stomach | Gastric cancer | 77/997 (7.72%) | 90 |
| **Colorectal** | **Colorectal cancer** | **58/669 (8.67%)** | **76** |
| Breast | Breast cancer | 55/1787 (3.08%) | 62 |
| Brain | Brain cancer | 56/559 (10.02%) | 59 |
| Brain | Pediatric Brain Tumor | 16/685 (2.34%) | 56 |
| Pancreas | Pancreatic cancer | 41/711 (5.77%) | 50 |
| Esophagus | Esophageal cancer | 37/633 (5.85%) | 47 |
| Skin | Melanoma | 26/100 (26%) | 46 |
| Uterus | Endometrial cancer | 22/250 (8.8%) | 30 |
| Blood | Malignant Lymphoma | 23/241 (9.54%) | 28 |
| Lung | Lung cancer | 25/402 (6.22%) | 24 |
| Prostate | Prostate cancer | 24/996 (2.41%) | 23 |
| Gall Bladder | Biliary Tract cancer | 14/310 (4.52%) | 18 |
| Head and neck | Thyroid cancer | 12/192 (6.25%) | 14 |
| Ovary | Ovarian cancer | 12/211 (5.69%) | 14 |
| Head and neck | Oral cancer | 10/178 (5.62%) | 12 |
| Kidney | Renal cancer | 11/995 (1.11%) | 12 |
| Mesenchymal | Soft Tissue cancer | 6/67 (8.96%) | 12 |
| Bladder | Bladder cancer | 9/236 (3.81%) | 10 |
| Uterus | Uterine cancer | 4/20 (20%) | 10 |
| Blood | Blood cancer | 9/255 (3.53%) | 9 |
| Cervix | Cervical cancer | 9/194 (4.64%) | 9 |
| Blood | Chronic Lymphocytic Leukemia | 6/510 (1.18%) | 6 |
| Blood | Chronic Myeloid Disorders | 4/136 (2.94%) | 4 |
| Bone | Bone cancer | 4/228 (1.75%) | 4 |

**Additional file 1: Table S2. Clinical and pathological characteristics of the COAD patients in TCGA**

| Variables | COAD (AJCC tumor stage) | | | |
| --- | --- | --- | --- | --- |
|  | Stage I | Stage II | Stage III | Stage IV |
| Sample size | 108 | 191 | 180 | 90 |
| Age |  |  |  |  |
| ≤52 | 15 | 19 | 40 | 18 |
| ＞52 | 93 | 172 | 140 | 72 |
| Gender |  |  |  |  |
| Male | 61 | 99 | 89 | 51 |
| Female | 47 | 92 | 91 | 39 |
| AJCC tumor pathologic PT |  |  |  |  |
| Low (T1/2) | 107 | 0 | 14 | 2 |
| High (T3/4) | 0 | 191 | 166 | 88 |
| AJCC nodes pathologic PN |  |  |  |  |
| Low (N0/1) | 108 | 191 | 112 | 45 |
| High (N2) | 0 | 0 | 68 | 45 |
| AJCC metastasis pathologic PM |  |  |  |  |
| M0 | 98 | 175 | 150 | 89 |
| M1 | 0 | 0 | 0 | 0 |
| Vascular invasion indicator |  |  |  |  |
| Yes | 17 | 26 | 45 | 39 |
| No | 75 | 145 | 112 | 42 |
| KRAS mutation |  |  |  |  |
| Yes | 0 | 10 | 7 | 11 |
| No | 1 | 8 | 11 | 11 |
| New tumor event after initial treatment |  |  |  |  |
| Yes | 0 | 3 | 4 | 3 |
| No | 5 | 10 | 9 | 4 |

**Additional file 1: Table S3. List of 9 CRC patient samples (for Fig. 1c and Figs. S2 and S3)**

| Sample No. | Patient No. | Histopathological Information |
| --- | --- | --- |
| 1 | S12-7036-K9 | descending colon, T3N0(2A) |
| 2 | S12-7710-K6 | descending colon, T3N0(2A) |
| 3 | S12-8394-K2 | cecum and sigmapod, T2N2 |
| 4 | S12-8629-K10 | ascending colon, T3N0(2A) |
| 5 | S12-8721-K12 | ascending colon, T3N0(2A) |
| 6 | S12-10060-K2 | ascending colon, T3N0(2A) |
| 7 | S12-10371-K6 | ascending colon, T3N0(2A) |
| 8 | S12-11243-K2 | ascending colon, T2N0(1) |
| 9 | S12-12799-K8 | rectosigmoid colon, T3N1b(3A) |

CRC patient samples were provided by Soonchunhyang University Hospital (South Korea).

**Additional file 1: Table S4. List of 4 CRC patients (for Fig. 1d)**

| Sample No. | Patient No. | Histopathological Information |
| --- | --- | --- |
| 1 | S10-261 | T2N2M0 (Stage 3) |
| 2 | S10-370 | T1N0M0 (Stage 1) |
| 3 | S10-960 | T3N1M0 (Stage 3) |
| 4 | S10-1285 | T3N1M0 (Stage 3) |

CRC patient samples were provided by Soonchunhyang University Hospital (South Korea).
